# Neuroimmune Dysregulation and the Role of IL-10 in Depression: A Systematic Review

**DOI:** 10.3390/brainsci16060548

**Published:** 2026-05-22

**Authors:** José Luis Cortes-Altamirano, Alfonso Alfaro-Rodríguez, Angélica González-Maciel, Beatriz Pérez-Guille, Rosa Eugenia Soriano-Rosales, Herlinda Bonilla-Jaime, Alberto Ávila-Luna, Antonio Bueno-Nava, Pedro Sánchez-Aparicio, Ana Lilia Dotor-Llerena

**Affiliations:** 1 Division of Basic Neurosciences, Instituto Nacional de Rehabilitación-LGII, Mexico City 14389, Mexicotato247@hotmail.com (A.Á.-L.);; 2 Department of Research, Universidad Estatal del Valle de Ecatepec, Ecatepec de Morelos 55210, Mexico; 3 National CONAHCyT Laboratory in Artificial Intelligence and Data Science (LNC-IACD), Ecatepec de Morelos 55210, Mexico; 4 Laboratory of Cellular and Tissue Morphology, Instituto Nacional de Pediatría, Secretaría de Salud, Mexico City 04300, Mexico; 5 Translational Research Center, Instituto Nacional de Pediatría, Mexico City 04300, Mexico; bettyepg@yahoo.com (B.P.-G.); resr62@yahoo.com.mx (R.E.S.-R.); 6 Department of Reproductive Biology, Universidad Autónoma Metropolitana, Mexico City 09340, Mexico; 7 Department of Pharmacology, Faculty of Veterinary Medicine, Universidad Autónoma del Estado de México, Toluca 50090, Mexico; 8 Division of Clinical Neurosciences, Instituto Nacional de Rehabilitación-LGII, Mexico City 14389, Mexico

**Keywords:** treatment-resistant depression, neuroinflammation, interleukin-10, neuroimmune dysregulation, cytokines, immunomodulation, inflammatory biomarkers

## Abstract

**Background:** Treatment-resistant depression (TRD) represents a major clinical challenge and is increasingly associated with persistent neuroinflammatory processes. Evidence suggests that dysregulation of the immune system, particularly the imbalance between pro-inflammatory and anti-inflammatory cytokines, contributes to poor therapeutic response. In this context, interleukin-10 (IL-10) has emerged as a key mediator in regulating the inflammatory response. **Objective:** To systematically analyze the evidence on neuroimmune dysregulation in depression, with an emphasis on TRD, and to evaluate the potential role of IL-10 as a biomarker and modulator of therapeutic response. **Methods:** A systematic review was conducted in accordance with PRISMA guidelines. Fourteen studies were included, comprising randomized clinical trials, longitudinal studies, a prospective cohort study, and exploratory designs. Methodological quality was assessed using the RoB 2 tool and complementary approaches. Data were integrated through a qualitative analysis focused on inflammatory biomarkers and clinical outcomes. **Results:** The studies consistently showed an association between elevated levels of pro-inflammatory cytokines, such as IL-6 and TNF-α, and the severity of depressive symptoms, as well as reduced response to conventional treatments. Immunomodulatory interventions, including ketamine, pentoxifylline, and minocycline, were associated with clinical improvement, particularly in patients with elevated baseline inflammation. IL-10 appears to be involved in counter-regulatory neuroimmune processes associated with inflammatory balance. **Conclusions:** Neuroinflammation plays a central role in TRD. IL-10 may serve as a relevant biomarker and a potential target for personalized therapeutic strategies informed by immune profiles, through modulation of neuroinflammatory pathways.

## 1. Introduction

Treatment-resistant depression (TRD) is classically defined as the absence of an adequate clinical response to at least two antidepressant drugs from different classes, administered at sufficient doses and for adequate duration [1]. This condition affects a significant proportion of patients with depressive disorders and is associated with poorer clinical prognosis, increased morbidity, and substantially higher healthcare costs. Emerging evidence has further emphasized TRD as a markedly heterogeneous and clinically burdensome condition, associated with substantial functional disability, increased healthcare utilization, and limited long-term therapeutic response, reinforcing the need for biologically informed, precision-based approaches to patient stratification and treatment development [2]. Despite the expanded range of antidepressant agents, a substantial proportion of patients continue to experience persistent symptoms, particularly cognitive deficits, highlighting the limitations of traditional pathophysiological models [3].

Historically, depression research has focused on the monoaminergic system; however, this approach does not fully explain the clinical complexity of TRD. A paradigm shift has moved toward integrative models that consider interactions among neurobiological and peripheral systems. Within this framework, the neuroimmune axis has emerged as a central component of TRD pathophysiology. Accumulating evidence suggests that immune dysregulation is not merely a consequence of chronic stress but also an active factor in the development and maintenance of depressive symptoms and treatment resistance [4]. Chronic low-grade neuroinflammation has been identified as a key mechanism linking immune alterations to synaptic dysfunction, altered neuroplasticity, and cognitive impairment [5].

However, this model is not without controversy. Although multiple studies have documented elevations in inflammatory markers in subgroups of patients with depression, the causal relationship between inflammation and TRD, as well as its therapeutic relevance, remains under debate. Clinical trials targeting inflammatory modulation have shown heterogeneous results, suggesting substantial biological variability among patients and highlighting the need for more precise stratification strategies [6].

In this context, immunomodulatory therapies have gained increasing interest as complementary approaches. Among them, interleukin-10 (IL-10), a key anti-inflammatory cytokine, has demonstrated neuroprotective and pro-cognitive effects in preclinical models, including modulation of microglial activation, restoration of synaptic plasticity, and improvement of hippocampus-dependent functions [7,8]. However, direct clinical evidence supporting IL-10-targeted therapeutic strategies in TRD remains limited. Current human studies primarily rely on observational analyses of inflammatory biomarkers and immune-associated depressive phenotypes rather than on interventions specifically designed to modulate IL-10 signaling. Consequently, the translational relevance of IL-10 in TRD should be considered emerging and exploratory.

The objective of this review is to analyze the available evidence on neuroimmune dysregulation and the role of IL-10 in depression, with particular emphasis on TRD and inflammation-associated depressive phenotypes.

## 2. Materials and Methods

A systematic review of the literature was conducted in accordance with PRISMA (Preferred Reporting Items for Systematic Reviews and Meta-Analyses) guidelines to identify and synthesize evidence on the role of the neuroimmune axis and interleukin-10 (IL-10) in TRD.

Eligible studies included those conducted in human populations diagnosed with major depressive disorder, particularly TRD, that evaluated neuroimmune mechanisms, inflammatory biomarkers, or immunomodulatory interventions involving IL-10 or related cytokine pathways. Randomized controlled trials, cohort studies, longitudinal studies, and clinical experimental designs were included. Only studies published in English were included. Animal studies, preclinical experimental studies, conference abstracts with insufficient data, narrative reviews, editorials, and studies lacking relevant neuroimmune or inflammatory outcomes were excluded. Gray literature was not systematically included. Diagnostic definitions of major depressive disorder (MDD) and TRD were accepted according to the criteria reported in each study. Pediatric and adolescent populations were not excluded a priori when studies provided mechanistic or clinically relevant evidence on inflammatory biomarkers, IL-10 signaling, or neuroimmune dysregulation in depressive disorders. Studies were required to report outcomes related to inflammatory biomarkers (such as IL-6, TNF-α, and IL-10), clinical response to treatment, or associations between immune dysregulation and depressive symptoms. Although the primary focus of this review was TRD, studies involving broader depressive populations or inflammation-associated depressive phenotypes were also included if they provided mechanistic or translational evidence relevant to neuroimmune dysregulation, cytokine imbalance, or IL-10-related signaling pathways that may be linked to treatment resistance. The literature search was performed in PubMed/MEDLINE, Web of Science, and Scopus databases, including studies published between January 2000 and March 2025, and was updated in January 2026 to incorporate newly published studies relevant to neuroimmune dysregulation and IL-10 signaling in depression and TRD. MeSH descriptors and free-text terms were used, combined with Boolean operators (AND, OR). The search strategy was adapted to each database. The optimized PubMed search strategy included the following terms: (“treatment-resistant depression” OR “TRD” OR “major depressive disorder”) AND (“interleukin-10” OR “IL-10”) AND (“neuroinflammation” OR “cytokines” OR “immune dysregulation” OR “microglia”). Equivalent search strategies were adapted for each database. Additionally, the reference lists of included studies were manually screened to identify further relevant articles.

The study selection process was conducted in four stages: identification, screening, eligibility, and inclusion. Duplicate records were manually identified and removed prior to independent screening of titles, abstracts, and full-text articles by two reviewers. Potentially eligible articles were assessed in full text, and those meeting the inclusion criteria were incorporated into the qualitative synthesis. Discrepancies were resolved by consensus.

Data extraction was performed independently by two reviewers using a standardized data collection framework. Extracted information included study design, sample size, population characteristics, type of intervention or exposure, duration of follow-up, inflammatory biomarkers assessed, and clinical outcomes. The primary outcomes of interest were changes in inflammatory biomarkers (particularly IL-10, IL-6, and TNF-α), clinical response to treatment, and associations between neuroimmune dysregulation and depressive symptom severity. Any discrepancies in data extraction were resolved by consensus among the reviewers.

The methodological quality and risk of bias of the included studies were assessed using validated tools according to study design. The Cochrane Risk of Bias tool version 2 (RoB 2) was applied exclusively to randomized controlled trials. Observational, longitudinal, and non-randomized studies were evaluated using NIH quality assessment tools appropriate to their study designs. Two reviewers independently performed the assessments, and disagreements were resolved through consensus. Studies were classified as having low, moderate, or high risk of bias.

Due to heterogeneity in study designs, populations, and assessed variables, a qualitative synthesis was conducted. The qualitative synthesis was organized according to predefined thematic domains, including neuroimmune dysregulation, inflammatory biomarkers, IL-10-associated signaling pathways, immunomodulatory interventions, and translational implications for treatment-resistant depression. The analysis focused on integrating clinical findings, emphasizing neuroimmune mechanisms, IL-10 signaling, and the translational relevance of these mechanisms in TRD.

Finally, a formal assessment of the certainty of evidence using the GRADE methodology and an evaluation of reporting bias were not performed. These omissions represent limitations of the present synthesis and should be considered when interpreting the findings. This limitation is acknowledged and discussed in the corresponding section of the manuscript.

## 3. Results

### 3.1. Study Selection and Characteristics of Included Studies

**Figure 1:**
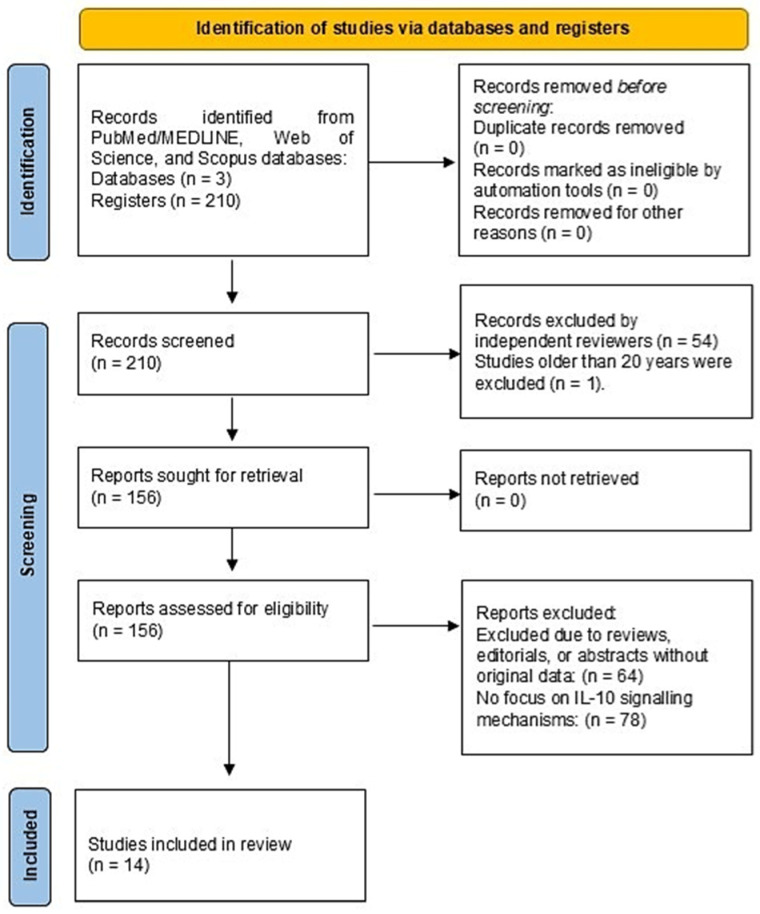
PRISMA 2020 flow diagram of the study selection process.

The studies reviewed comprised both formally defined TRD cohorts and broader depressive populations characterized by inflammatory activation, metabolic comorbidity, cytokine dysregulation, or experimentally induced depressive symptoms. The study selection process followed PRISMA 2020 guidelines. A total of 210 records were initially identified. After title and abstract screening, 54 studies were excluded for not meeting the inclusion criteria; only one article was excluded due to the time restriction (2000–2025). Consequently, 156 articles were assessed in full text. Of these, 141 were excluded: 64 were reviews, editorials, or abstracts without original data, and 78 did not address mechanisms related to IL-10 signaling. In total, 14 studies met the inclusion criteria and were included in the qualitative synthesis. The complete selection process is shown in the PRISMA flow diagram (Figure 1) The PRISMA Check list is found in Table S1. Included studies were grouped into the following categories: pharmacological immunomodulatory interventions (e.g., ketamine, pentoxifylline, minocycline), nutritional and metabolic approaches, neuromodulatory interventions, observational biomarker studies, and inflammation-associated depressive models.

Randomized clinical trials primarily evaluated adjunctive or alternative therapies with immunomodulatory potential, such as pentoxifylline, ketamine, minocycline, and nutritional supplementation. Among the included studies, most of them directly quantified IL-10 levels as part of inflammatory biomarker panels, whereas a smaller subset inferred IL-10-related mechanisms indirectly through broader neuroimmune or cytokine-associated findings. Longitudinal and observational studies focused on characterizing the inflammatory profile and its evolution during disease progression or treatment. Overall, methodological quality ranged from moderate to high. Clinical trials showed a lower risk of bias, whereas non-randomized studies had inherent limitations, including a lack of control and reduced capacity for causal inference (Table 1).

Findings from this review support an association between pro-inflammatory pathway activation and both the severity of depressive symptoms and reduced antidepressant response. Studies evaluating immunomodulatory interventions showed that cytokine modulation is associated with clinical improvement, particularly in patients with elevated inflammatory profiles. In this context, IL-10 emerges as a key component of immune balance, either directly measured or inferred, highlighting its dynamic role as a counter-regulatory mediator whose dysfunction may contribute to persistent neuroinflammation and treatment resistance (Table 2).

### 3.2. Neuroimmune Dysregulation in Depression and TRD

The conceptualization of TRD has evolved to recognize immune dysregulation as a key component of its pathophysiology. Numerous studies support the notion that abnormal activation of the neuroimmune axis, characterized by neuroinflammatory processes, plays a significant role in depressive symptoms, particularly in patients whose symptomatology appears driven by inflammatory mechanisms. Nevertheless, the extent and clinical relevance of immune dysregulation in depression remain incompletely understood, and uncertainties persist regarding its specific role in treatment resistance [23,24,25,26].

Inflammation-induced exacerbation worsens key symptoms, such as anhedonia, particularly in individuals with elevated inflammatory profiles [10]. Additionally, longitudinal and cohort studies suggest that elevated pro-inflammatory cytokines may precede and predict the development of depressive symptoms, supporting a causal role of immune activation in depression pathogenesis [22].

Clinically, several trials have shown that modulation of the inflammatory response is associated with symptom improvement. For example, pentoxifylline as an adjunctive therapy significantly reduced depressive symptoms and pro-inflammatory cytokine levels [11,17]. Similarly, studies in TRD patients treated with ketamine showed that reductions in inflammatory cytokines correlate with antidepressant response, particularly in subgroups with elevated baseline inflammation [15]. These findings suggest the existence of an inflammation-driven subtype of depression with direct implications for treatment resistance.

### 3.3. Role of IL-10 in Neuroinflammatory Modulation

Interleukin-10 was identified as a key factor in regulating inflammatory balance. Several studies analyzing cytokine profiles reported alterations in both pro-inflammatory and anti-inflammatory mediators, including IL-10, suggesting immunoregulatory dysfunction rather than simple inflammatory activation [16,19].

IL-10 exhibited a dynamic behavior associated with both baseline inflammatory status and treatment response. Patients with greater immune activation showed altered IL-10 levels and increased susceptibility to inflammation-induced symptom changes [9]. In intervention studies, cytokine modulation following treatments such as ketamine was characterized by changes in IL-10, suggesting its involvement in resolving inflammatory responses [15]. However, other studies reported no significant changes in IL-10 despite clinical improvement, suggesting that its role may depend on the specific pathophysiological context [9,13]. Overall, these findings support the hypothesis that TRD is associated with an imbalance between pro-inflammatory cytokines (e.g., IL-6 and TNF-α) and IL-10-mediated anti-inflammatory mechanisms, whose dysfunction may contribute to persistent neuroinflammation.

## 4. Discussion

The findings of this systematic review reinforce the notion that TRD should be understood as a distinct biological phenotype characterized by persistent dysregulation of the neuroimmune axis. Sustained activation of inflammatory pathways is associated not only with symptom severity but also represents a central mechanism contributing to therapeutic resistance [10,21]. This perspective aligns with theoretical models linking inflammation to the transition from “sickness behavior” to clinical depression, mediated by chronic activation of central immune cells [23,27,28,29]. The inclusion of broader depressive and inflammation-related populations allowed the integration of mechanistic evidence potentially relevant to TRD, although direct extrapolation to treatment-resistant populations should be undertaken cautiously. Within this framework, TRD may be interpreted as a condition in which endogenous regulatory mechanisms are insufficient to counteract persistent neuroinflammation [30].

Integrating the neuroimmune axis into TRD pathophysiology represents a paradigm shift from the classical monoaminergic hypothesis to a more complex systemic model. Evidence indicates that a significant proportion of patients with major depressive disorder exhibit chronic inflammatory states contributing to treatment resistance [15,31]. Mechanistically, this state is characterized by sustained increases in pro-inflammatory cytokines such as IL-6 and TNF-α, which promote microglial activation, alter synaptic plasticity, and disrupted neural circuits involved in mood regulation [24,25]. In this context, IL-10 emerges as a key regulator of inflammatory balance, acting as a physiological brake on immune responses. However, findings suggest that in TRD, this counter-regulatory mechanism may be insufficient or dysfunctional, favoring the persistence of a pro-inflammatory microenvironment [16,19].

A particularly relevant finding is the apparent “contradiction” of IL-10. Although it has well-established anti-inflammatory properties, its behavior in clinical studies is not uniform. In some cases, elevated IL-10 levels are associated with persistent inflammatory states, suggesting an ineffective compensatory response rather than effective protection [20,22]. In other scenarios, its reduction following interventions such as ketamine administration has been interpreted as restoration of immune homeostasis. This variability indicates that the role of IL-10 is highly context-dependent.

**Figure 2:**
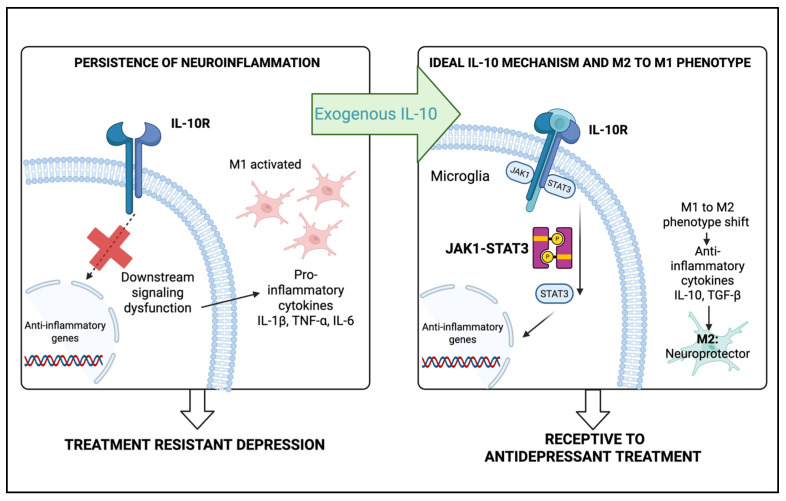
Proposed role of IL-10 signaling in neuroimmune dysregulation associated with TRD. IL-10 binding to the IL-10 receptor (IL-10R) activates the JAK1/STAT3 pathway, promoting anti-inflammatory responses and modulating microglial activation. Dysfunction or insufficiency of IL-10-mediated signaling may contribute to persistent neuroinflammation, impaired synaptic plasticity, and a sustained pro-inflammatory microenvironment. The figure illustrates the proposed balance between pro-inflammatory microglial phenotypes (M1-like) and regulatory/neuroprotective phenotypes (M2-like) within the neuroimmune context of depression.

From a mechanistic perspective, this contradiction may be explained by alterations in IL-10 intracellular signaling. The effect of this cytokine relies not only on its concentration but also on the integrity of downstream pathways, particularly the JAK1/STAT3 axis [32]. Defects in receptor sensitivity or STAT3 activation may limit IL-10’s capacity to induce a neuroprotective microglial phenotype, analogous to the M2 state, thereby perpetuating inflammation [33]. Consequently, therapeutic strategies that aim solely at increasing IL-10 levels may be insufficient without addressing the functionality of the signaling pathways. These mechanistic interpretations are primarily supported by preclinical and translational evidence, whereas direct clinical evidence for TRD remains limited and warrants further validation (Figure 2).

Clinically, the results support the conceptualization of TRD as an inflammatory subtype of depression, with direct implications for patient stratification. Patients with elevated baseline inflammation tend to respond better to immunomodulatory therapies such as pentoxifylline, minocycline, or ketamine [11,15,17]. Accordingly, immunological interventions should preferentially target an “inflamed biotype,” identified through markers such as elevated C-reactive protein or increased IL-6 and TNF-α levels [34,35]. Lack of proper stratification may explain the failure of some anti-inflammatory trials that included patients without significant immune activation [36].

Beyond experimental evidence, ketamine and intranasal esketamine are the most clinically translated examples of rapidly acting interventions for TRD. Although their antidepressant effects are primarily linked to glutamatergic modulation and synaptic plasticity, growing evidence suggests that immune and inflammatory mechanisms may also contribute to therapeutic response, particularly in patients with elevated baseline inflammatory profiles. Clinical experience with intranasal esketamine has reinforced its relevance in TRD management while highlighting the biological heterogeneity underlying treatment responsiveness. However, the immunomodulatory contribution of ketamine remains incompletely understood and should be interpreted within a multifactorial neurobiological framework [37].

In this context, IL-10 may be more informative when assessed as part of a composite immune signature rather than as an isolated biomarker. Integrating IL-10 with additional inflammatory mediators, such as IL-6, TNF-α, and C-reactive protein, may improve identification of inflammation-associated depressive phenotypes and facilitate biologically informed stratification strategies in TRD. Future precision psychiatry approaches will likely require multidimensional biomarker panels that combine inflammatory, metabolic, and neuroplasticity-related indicators.

From a translational perspective, these findings open new avenues for personalized therapeutic strategies. The synergy between traditional antidepressants and therapeutic strategies targeting IL-10-related pathways may represent an innovative approach to addressing both affective symptoms and persistent cognitive deficits associated with TRD [29]. However, the clinical application of IL-10 faces challenges, including limited penetration across the blood–brain barrier and the risk of systemic immunosuppression [38].

Novel delivery approaches, such as intranasal administration, and the development of small-molecule mimetics targeting the JAK/STAT pathway represent promising strategies [39]. Additionally, functional evaluation of signaling pathways, beyond cytokine level measurements, may improve the accuracy of patient stratification.

### Limitations

Despite the consistency of findings, this review presents relevant limitations. Heterogeneity in study designs, populations, and methodologies limits direct comparability. Additionally, IL-10 was not uniformly assessed across studies, requiring inference in some cases. Methodological limitations identified in risk-of-bias assessments, along with small sample sizes in several studies, should be considered when interpreting the results. Additionally, a formal certainty-of-evidence assessment using the GRADE methodology was not conducted, limiting the overall strength and interpretability of the synthesized evidence. Several included studies had relatively small sample sizes, heterogeneous clinical populations, and older methodological designs, potentially affecting the robustness and generalizability of the findings. Furthermore, IL-10 was frequently evaluated as a secondary, exploratory, or incidental biomarker within broader inflammatory panels rather than as a primary endpoint, limiting the strength of IL-10-specific mechanistic conclusions. This systematic review was conducted according to PRISMA 2020 guidelines; however, the protocol was not prospectively registered in PROSPERO or the Open Science Framework (OSF). This should be considered a methodological limitation.

## 5. Conclusions

Overall, the evidence suggests that TRD can be understood as a condition characterized by persistent dysregulation of the neuroimmune axis, in which the imbalance between pro-inflammatory and anti-inflammatory signals plays a central role. In this context, IL-10 emerges as a key immune modulator whose dysfunction may contribute to symptom chronicity and therapeutic resistance. Although its clinical application requires further validation, its integration into precision medicine models represents a promising pathway toward developing more effective, personalized therapeutic strategies for depression.

## Figures and Tables

**Table 1:** Characteristics and quality assessment of the included studies.

Author	Study Design	Assessment Tool	Quality	Main Limitations of the Study
Venable et al., 2025. [9]	Placebo-controlled Clinical trial	RoB 2	Moderate - high	Blinding is missing
Savitz J et al., 2025. [10]	Double blinded randomized clinical trial	RoB 2	High	Sample size and subjective outcomes
Merza Mohamm et al., 2024. [11]	Double blinded randomized clinical trial	RoB 2	High	Allocation concealment and intention-to-treat analysis
Nettis MA et al., 2023. [12]	Mixed-methods clinical intervention study	NIH	Moderate	Mixed methods and possible confusion in biomarkers
Vaghef-ehrabani et al., 2023. [13]	Randomized clinical trial	RoB 2	Moderate	Dietary adherence, cointerventions and missing blinding
Langhein M et al., 2022. [14]	Longitudinal pilot study	NIH	Moderate - low	Small sample size and absence of a robust control group
Zhou Y et al., 2021. [15]	Longitudinal clinical study	NIH	Moderate	Clinical heterogeneity and absence of a robust control group
Pérez-Sánchez G et al., 2018. [16]	Open follow-up study	NIH	Moderate - low	Open design and no blinding
El-Haggar et al., 2018. [17]	Double blinded randomized clinical trial	RoB 2	High	Small sample for being proof-of-concept
Brunoni AR et al., 2018. [18]	Clinical trial	RoB 2	Moderate - high	Biomarkers as secondary outcome
Zou W et al., 2018. [19]	Longitudinal clinical study	NIH	Moderate	Causal inference limited Missing randomization
Song C et al., 2009 [20]	Comparative intervention study	RoB 2	Moderate - low	Unclear method of assignment
Hernández ME et al., 2008. [21]	Follow-up longitudinal study	NIH	Moderate	No control randomized group
Wichers MC et al., 2006. [22]	Prospective cohort study	NIH	Moderate - high	Comparability between groups

**Table 2:** Included studies regarding neuroinflammatory biomarkers and the role of IL-10 in treatment-resistant depression.

Author and Year	Population Characteristics	Intervention or Exposition	Biomarkers Evaluated	Main Findings	Role of IL-10 (Direct or Inferred)	Relevance in TRD
Venable et al., 2025. [9]	n = 43; rural adults, mild to moderate symptoms of depression and anxiety	Blueberry supplementation vs. placebo	IL-1β, IL-6, TNF-α, IFN-γ, IL-10, CRP, metabolomics	It improved depressive and anxiety symptoms, but without significant changes in inflammatory cytokines, suggesting a clinical effect not clearly mediated by peripheral inflammation.	Measured directly; its stability suggests that IL-10 was not the primary mediator in this context, although it does not rule out a basal regulatory role.	Indirect relevance; it suggests that not all patients with TRD exhibit detectable inflammatory changes.
Savitz J et al., 2025. [10]	n = 70; adults, with subgroups based on baseline inflammation (low/high)	Inflammatory challenge with LPS vs. placebo	IL-6, IL-10, TNF, CRP, SHAPS, MADRS	Subjects with high baseline inflammation showed a greater increase in anhedonia and IL-6 following LPS administration, supporting a phenotype that is biologically sensitized to inflammation.	Measured directly; no significant differences were observed, suggesting that an inadequate anti-inflammatory response to the immune challenge may contribute to the persistence of the pro-inflammatory state.	High; identifies an inflammatory subgroup that is more susceptible to persistent symptoms, particularly anhedonia.
Merza Mohamm et al., 2024. [11]	n = 100; adults being treated with SSRIs	Citalopram + pentoxifylline vs. citalopram + placebo	IL-1β, TNF-α, PCR, IL-6, IL-10, serotonin, BDNF	Pentoxifylline increased response and remission rates and reduced pro-inflammatory markers, supporting its role as an immunomodulatory target in depression.	Measured directly; is integrated as restoration of the pro- and anti-inflammatory balance induced by the treatment.	High; supports the notion that inflammatory profiles can identify candidates for adjuvant strategies in cases of treatment resistance.
Nettis MA et al., 2023. [12]	n = 39; patients con TRD	Adjuvant Minocycline vs. placebo	CRP, TNF, IL-10, metabolites of the kynurenine pathway	The KYN/TRP coefficient was associated with CRP and IL-10 y con suicidal ideation; minocycline did not clearly alter metabolites over 4 weeks, although it showed a tendency to reduce suicidal ideation.	Measured directly; IL-10 was associated with activation of the kynurenine pathway, suggesting a dual compensatory/homeostatic role in persistent inflammation.	Very high; direct evidence in TRD with low-grade inflammation and supports an immune-related subtype.
Vaghef-ehrabani et al., 2023. [13]	n = 45; women with obesity and depression	Insulin + Calorie restriction vs. maltodextrin + calorie restriction	LPS, zonulin, TNF-α, IL-10, MCP-1, TLR-4, hs-CRP, BDNF	There were no significant differences in HDRS or inflammatory biomarkers, suggesting that short-term prebiotic intervention was not sufficient to reverse the inflammatory phenotype.	Measure directly; the lack of changes suggests that IL-10 may be resistant to short-term dietary interventions when obesity and depression coexist.	Moderate; relevant for metabolic-inflammatory subgroups with a higher potential risk of non-response.
Langhein M et al., 2022. [14]	n = 20; adults with depression	Ketamine; peripheral inflammation y free-water imaging before/after	Peripheral inflammatory markers + white matter neuroimaging	Suggested an association between peripheral inflammation and microstructural changes, as well as the potential predictive value of inflammation for the response to ketamine.	Inferred; IL-10 is thought to be part of the counter-regulatory axis, the effectiveness of which may modulate the relationship between peripheral inflammation and the immediate response.	High; supports the use of biomarkers for patient stratification in rapid-acting therapies for treatment-resistant depression.
Zhou Y et al., 2021. [15]	n = 66 TRD + 60 controls; subgroup con dolor	Six ketamine infusions	19 cytokine panel, including IL-6, TNF-α, IL-10, GM-CSF	Patients with pain exhibited greater baseline inflammation and a better response/remission with ketamine; this improvement was accompanied by a broad reduction in cytokines, including IL-10.	Measured directly; its post-treatment decrease suggests that IL-10 may reflect a baseline compensatory activation that normalizes as inflammation resolves.	Very high; direct evidence in TRD and highlights the value of identifying inflammatory subgroups associated with pain.
Pérez-Sánchez G et al., 2018. [16]	n = 22 patients + 18 controls; adolescents	Fluoxetine during 8 weeks	IL-2, IFN-γ, IL-1β, TNF-α, IL-6, IL-12, IL-15, IL-4, IL-5, IL-13, IL-1Ra, IL-10	Depressed adolescents showed elevated levels of multiple cytokines; during treatment, several pro-inflammatory mediators decreased, while IL-4 and IL-5 increased, although IL-10 was not among the most notable changes.	Measured directly; its less pronounced response suggests that anti-inflammatory regulation may be partial or dependent on the stage of treatment.	Moderate; useful for understanding early inflammation, although not a direct cause of TRD.
El-Haggar et al., 2018. [17]	n = 80; adults with moderate depression	Escitalopram + pentoxifylline vs. escitalopram + placebo	TNF-α, IL-6, IL-10, BDNF, 8-OHdG, serotonin	Pentoxifylline resulted in greater clinical improvement and a reduction in TNF-α, IL-6, and IL-10, along with an increase in BDNF and serotonin.	Measured directly; The decrease in IL-10, along with pro-inflammatory cytokines, suggests the normalization of a previously activated compensatory response.	High; supports the hypothesis of immunomodulation as an adjuvant strategy in patients with a poor response.
Brunoni AR et al., 2018. [18]	n = 236; adults in multiple treatment arms	tDCS vs. escitalopram vs. placebo	NGF, BDNF, GDNF, IL-1β, IL-6, IL-8, IL-10, IL-12p70, IL-18, IL-33, TNF-α, sTNFr1/2	In general, biomarkers did not predict response; IL-10 levels decreased over time regardless of group or clinical response.	Measured directly; IL-10 functioned more as a nonspecific dynamic marker than as a differential predictor of response.	Moderate; suggests that isolated IL-10 may not distinguish TRD subgroups without being combined with other markers.
Zou W et al., 2018. [19]	n≈80; naïve patients	Baseline status without treatment	IL-1β, IL-6, IL-8, TNF-α, IL-10, TGF-β1	Patients had elevated levels of IL-1β, TNF-α, and IL-10 compared with controls, supporting the notion of complex immune activation from the early stages of the disease.	Measured directly; elevated IL-10 suggests a compensatory response to pro-inflammatory activation rather than effective protection.	High—indirect; indicates an inflammatory subtype that is likely to progress to non-response.
Song C et al., 2009 [20]	n = 95 + 30 controls; adults	Electroacupuncture vs. fluoxetine vs. placebo	IL-1β, IL-10, cytokines Th1/Th2	Elevated levels of IL-1β and decreased levels of IL-10 were observed in depression; the treatments tended to correct the pro-inflammatory/anti-inflammatory and Th1/Th2 imbalances.	Measured directly; low IL-10 levels served as an indicator of a loss of anti-inflammatory tone, consistent with sustained neuroinflammation.	High—indirect; a useful model for conceptualizing resistance associated with counter-regulatory failure.
Hernández ME et al., 2008. [21]	n = 31; adults undergoing long-term treatment	Treatment with SSRIs for 52 weeks	IL-1β, IL-10, IL-2, IFN-γ, IL-4, IL-13, cortisol	Before treatment, IL-10 and IL-4/IL-13 levels were elevated; with long-term follow-up, Th2 cytokines and IL-10 decreased, and only a partial restoration of the HPA-immune axis persisted.	Measured directly; elevated baseline IL-10 levels and their delayed decline suggest prolonged compensatory activation and incomplete resolution.	Moderate—high; illustrates that immune normalization can be slow and incomplete, a finding consistent with partial resistance.
Wichers MC et al., 2006. [22]	n = 16; patients receiving IFN-α without initial depression	Basal immune activation as an exposure	sIL-2R, IL-6, IL-10	High baseline levels of immune activation, including IL-10, predicted the subsequent onset of depression during IFN-α treatment, supporting a causal role for the immune system.	Measured directly; elevated IL-10 is interpreted as a marker of prior immune activation and biological vulnerability, not necessarily of effective protection.	High; emphasizes that a baseline inflammatory phenotype may precede depression and potentially contribute to TRD.

## Supplementary Materials

The following supporting information can be downloaded at: https://www.mdpi.com/article/10.3390/brainsci16060548/s1, Table S1: PRISMA 2020 Checklist.

## Abbreviations

The following abbreviations are used in this manuscript:

BDNF	Brain-Derived Neurotrophic Factor
CRP	C-Reactive Protein
HDRS	Hamilton Depression Rating Scale
HPA	Hypothalamic–Pituitary–Adrenal Axis
IFN-α	Interferon Alpha
IL	Interleukin
IL-1β	Interleukin-1 Beta
IL-2	Interleukin-2
IL-6	Interleukin-6
IL-10	Interleukin-10
JAK	Janus Kinase
KYN	Kynurenine
KP	Kynurenine Pathway
LPS	Lipopolysaccharide
MADRS	Montgomery–Åsberg Depression Rating Scale
MDD	Major Depressive Disorder
RCT	Randomized Controlled Trial
SSRI	Selective Serotonin Reuptake Inhibitor
tDCS	Transcranial Direct Current Stimulation
TNF-α	Tumor Necrosis Factor Alpha
TRD	Treatment-Resistant Depression
